# Substrate Topography Determines Neuronal Polarization and Growth *In Vitro*

**DOI:** 10.1371/journal.pone.0066170

**Published:** 2013-06-13

**Authors:** Liesbeth Micholt, Annette Gärtner, Dimiter Prodanov, Dries Braeken, Carlos G. Dotti, Carmen Bartic

**Affiliations:** 1 Life Science Technologies, Imec, Leuven, Belgium; 2 Solid State Physics and Magnetism Section, University of Leuven, Leuven, Belgium; 3 VIB Center for the Biology of Disease, Leuven, and Center for Human Genetics, University of Leuven, Leuven, Belgium; 4 Centro de Biología Molecular “Severo Ochoa”, CSIC/UAM, Madrid, Spain; 5 Environment Health and Safety, Imec, Leuven, Belgium; Universidade Federal do ABC, Brazil

## Abstract

The establishment of neuronal connectivity depends on the correct initial polarization of the young neurons. *In vivo*, developing neurons sense a multitude of inputs and a great number of molecules are described that affect their outgrowth. *In vitro,* many studies have shown the possibility to influence neuronal morphology and growth by biophysical, i.e. topographic, signaling. In this work we have taken this approach one step further and investigated the impact of substrate topography in the very early differentiation stages of developing neurons, i.e. when the cell is still at the round stage and when the first neurite is forming. For this purpose we fabricated micron sized pillar structures with highly reproducible feature sizes, and analyzed neurons on the interface of flat and topographic surfaces. We found that topographic signaling was able to attract the polarization markers of mouse embryonic neurons -N-cadherin, Golgi-centrosome complex and the first bud were oriented towards topographic stimuli. Consecutively, the axon was also preferentially extending along the pillars. These events seemed to occur regardless of pillar dimensions in the range we examined. However, we found differences in neurite length that depended on pillar dimensions. This study is one of the first to describe in detail the very early response of hippocampal neurons to topographic stimuli.

## Introduction

In vitro, neuronal polarization –i.e. the transition from a newly born round cell to an electrically active and fully interconnected adult neuron- takes place in five distinct steps. In stage 1 -shortly after plating- several thin filopodia are formed. Some hours later several minor processes -immature neurites- are generated during stage 2. In stage 3, 12h post plating, one neurite rapidly extends to form the axon, and all the neurites mature over the next week during stage 4. Finally dendrites and axon become synaptically functional in stage 5 [1], [2]. The fact that polarization occurs in the homogenous environment of the tissue culture dish has led to the notion that this is an autonomous, intrinsic, process [3], [4]. Recent work revealed that one such mechanism is the presence of an N-cadherin cluster in one pole of the cell in the immediately post-mitotic neuron, from which a first neurite subsequently emerges [5], [6]. The first neurite afterwards becomes the axon [2], [7]. In agreement with an essential role of the N-cadherin crescent in cell polarization, the ectopic exposure to exogenous N-cadherin was shown sufficient to favor the formation of the first bud at the new site [5]. This last result indicates that, despite their intrinsic predisposition to polarize at a given place (i.e. the pole with clustered N-cadherin), a newborn neuron has the nominal capacity to allow neurite formation everywhere in the sphere. This possibility could be of great value in nerve regeneration scenarios.

Numerous *in vitro* studies have shown that different types of structured surfaces can have a strong influence on neuronal growth and morphology [8]–[11]. This is also true *in vivo*, where cells are not only influenced by chemical signaling but also by topographic structures, as they provide mechanical support and guidance for growth and differentiation. Examples of *in vivo* guiding topography are the already formed glial processes and pre-existing axons, along which new axons migrate to establish connectivity [12]–[15]. Many studies have sought to explain how cellular and, more specifically, neuronal morphology are determined by topography [10], [16]–[19]. An important observation was that neurons sense isotropic micro-fabricated pillar surfaces, since they align to the pillar geometry. This can be explained by the tendency of neurites to follow existing contacts, but if a new contact -i.e. pillar- is nearby, they extend to contact the new location resulting in highly aligned and branched neuronal arbors [8], [11], [20]. All of these studies focused on neurons in stage 3 and further, when axon and dendrite identities were already defined. The study of the role of topography in relation to the generation of the first neurite is highly relevant since the position of the first neurite defines the axis of migration of neurons, and this is important for the proper organization of the brain [5]–[7].

In recent years different microstructures have been used to influence neuronal growth and behavior. In general, these used either anisotropic [19], [21] – e.g. fibers and grooves- and isotropic [8], [22] – e.g. pillars, holes and nanorough surfaces- topographic stimuli. All of them have been shown to affect neuronal morphology. However, whether any of these has a more pronounced effect on axonal development and growth than others is not clear. When neurons are plated on grooved substrates they can either align according to the direction of the groove (‘contact guidance’) or orient perpendicular to the grooves, generally referred to as ‘perpendicular contact guidance’ [19]. With the use of interrupted microstructures (holes/pillars) this type of contact guidance is more readily realized [10], [11]. In a recent publication Fozdar *et al.* [16] compared different shapes (lines and holes of (i) 300 nm and (i) 2 µm) for their ability to attract axons. The results show that for both dimension sizes, holes –or in general, interrupted features- are a more potent topographic cue to attract axonal specification, with over 70% of neurons extending to the holes vs grooves. Also *in vivo* the existence of ‘guidepost’ cells, which are specific cells located at discrete locations during embryonic development that serve as ‘stepping stones’ during axon pathfinding, is described in literature and has been shown in invertebrates [11], [23] and it has been suggested that this mechanism is conserved throughout vertebrates and perhaps even mammals [24].

In this work we investigated how neurons polarize in response to topographic stimuli and how this affects the outgrowth behavior. To this end we microfabricated surfaces having particular, highly reproducible, topographic features, and demonstrated their ability to influence the early phases of neuronal polarization, notably the formation of the first neurite, axonal differentiation and growth.

## Results

### Substrate Layout

**Figure 1:**
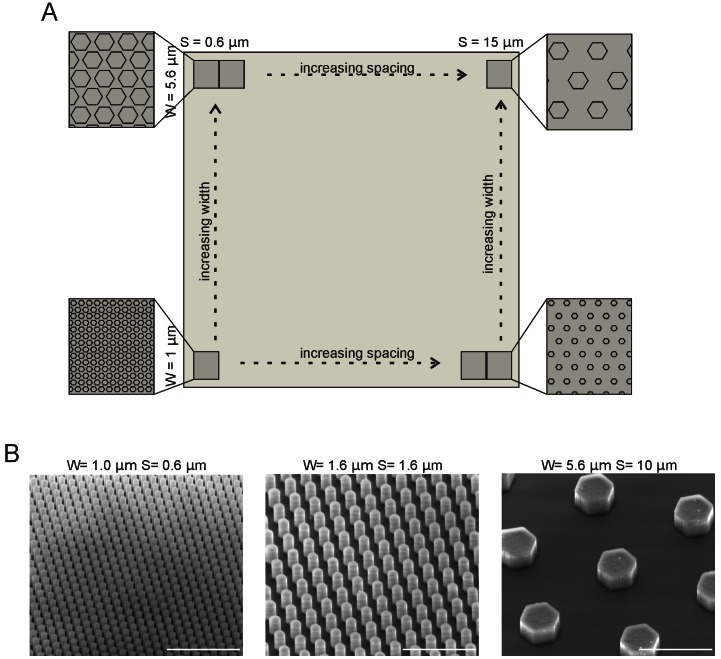
Substrate Lay-out. (**A**) The substrate consisted of individual areas decorated with pillars of different dimensions. The pillar width ranged from 1–5.6 µm (1, 1.2, 1.4, 1.6, 1.8, 2, 2.4, 2.8, 4, 5.6 µm) in the vertical direction, while the spacing ranged from 0.6–15 µm (0.6, 0.8, 1, 1.2, 1.4, 1.6, 1.8, 2.0, 2.4, 3.2, 4, 5, 7, 10, 15 µm) and the height was kept constant at 3 µm. (**B**) Scanning electron microscopy images of different width and spacing of pillars. Left is W = 1 µm, S = 1 µm, middle is W = 1.6 µm, S = 1.6 µm, right is W = 5.6 µm, S = 10 µm. Scale bars are 10 µm.

The substrates used for the cell cultures were diced from silicon wafers and consisted of different areas decorated with pillars having different diameters (1, 1.2, 1.4, 1.6, 1.8, 2, 2.4, 2.8, 4, 5.6 µm), different spacings between pillars (0.6, 0.8, 1, 1.2, 1.4, 1.6, 1.8, 2.0, 2.4, 3.2, 4, 5, 7, 10, 15 µm) and a height of 3 µm (Fig. 1A). In this way a matrix of different pillar configurations was created on a single support. Scanning electron microscopy images of areas with different pillar dimensions are shown in Fig. 1B. Between beds of pillars with different diameters, there is a flat border area (Fig. 1A, vertical direction). Cells that attached to the interface between the pillar array and the flat area could thus sense both the flat and the pillar-decorated surface and were important for our analysis. The substrates were coated with poly-L-lysine (PLL) before plating primary hippocampal neurons.

To assess the response to various pillar configurations, we analyzed the polarity parameters of freshly dissociated primary hippocampal neurons that settled at the border between the flat substrate and pillars. Before plating, neurons were kept in suspension for 2 hours to allow for the regeneration of surface molecules that may have been destroyed during the trypsin treatment used for the cell dissociation.

### Effect of Pillar Contact on First Sprout and Golgi Position

**Figure 2:**
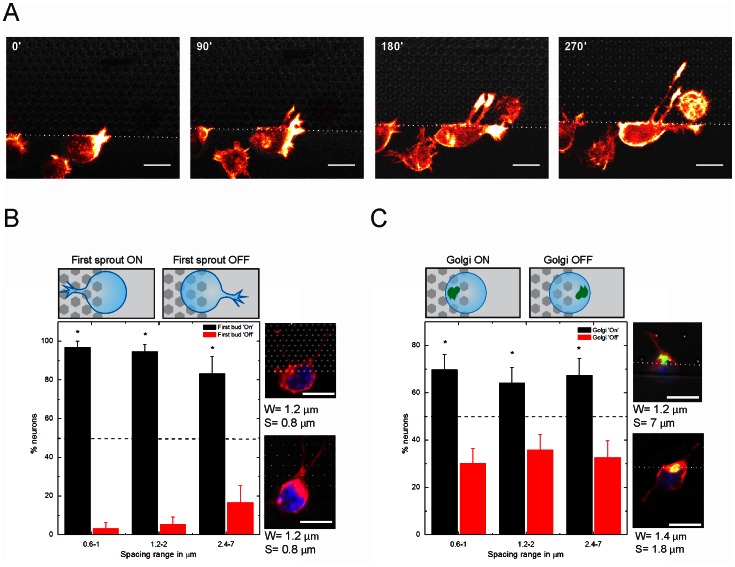
Analysis of first bud and Golgi-centrosome complex position. (**A**) Time lapse of neurons expressing the F-actin probe GFP-UtrCH, located on the edge of a pillar bed. Scale bar is 10 µm. (**B**) Analysis of the first sprout positioned towards (ON) or away (OFF) from the pillars. Dashed line indicates the 50% level. Examples of cells with first sprout ON are shown at the right (**C**) Analysis of the Golgi positioned towards (ON) or away (OFF) from the pillars. Examples of cells with Golgi ON are shown at the right. Scale bars are 10 µm. *, significantly different from random using X^2^ test (p<0.05). Blue, Nuclei (Hoechst), Green, Golgi apparatus (GPP130), red, microtubules (tuj-1), grey, substrate. For (B), 0.6–1 n = 31, 1.2–2 n = 37, 2.4–7 n = 18. For (C), 0.6–1 n = 53, 1.2–2 n = 53, 2.4–7 n = 43.

Firstly, we assessed the effect of the topography on the initial outgrowth of stage 1 neurons. Since filamentous actin is very dynamic in the initial stages of neuronal polarization in the outgrowing neurites, we performed time lapse imaging using the F-actin probe GFP-UtrCH (Calponin Homology domain of Utrophin) [25], [26]. These experiments revealed that the presence of a topographic signal determined the position of the first sprout similar to the situation illustrated in Fig. 2A, where even two sprouts simultaneously appeared on the “pillar-side” of the cell. We quantified the first bud distribution with respect to the pillar contact, taking into account cells with one extension (or multiple extensions that were all facing the pillars). The topographic features were grouped according to pillar spacing in dense (0.6–1 µm), intermediate (1.2–2 µm) and sparse (2.4–7 µm). Fig. 2B shows that the first sprout was preferentially located on the pillar-containing region, regardless of the inter-pillar spacing (96.8±3.2%, 94.6±5.4% and 83.3±16.7% for a spacing range of 0.6–1 µm, 1.2–2 µm and 2.4–7 µm, respectively).

Next, we analyzed whether the intracellular polarization of the Golgi apparatus was also influenced by the pillar proximity. For this, neurons with multiple minor neurites were also taken into account. The Golgi-centrosome complex is generally highly co-localized with the first neurite and the axon, and was therefore taken here as a simple indicator of neuronal polarization [7], [27], [28]. For quantification, the Golgi apparatus position was registered as being ON, when located towards the pillar topography, OFF when oriented towards the flat. In the current experimental set-up, the Golgi-centrosome complex position was enriched at the side of pillar contacts (Fig. 2C). Similar to what has been described in other experimental conditions [28]–[30], the Golgi-centrosome complex position was also found here to be at the base of the first neurite. Even at this very early stage (2–4 hours in culture) in about 67% of all cases, the Golgi-centrosome was oriented towards the pillar contacts for inter-pillar spacings between 0.6 and 2 µm (69.8±6.3%, 64.2±6.6% and 67.4±7.1% for a spacing range of 0.6–1 µm, 1.2–2 µm and 2.4–7 µm, respectively). These experiments show that topographic cues are robust inductors of neuronal polarization.

### Effect of Pillar Contact on N-cadherin Crescent

**Figure 3:**
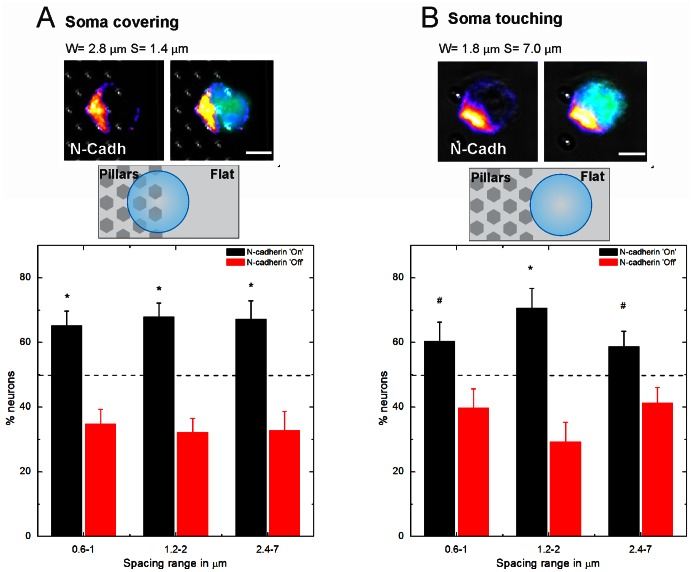
Effect of pillar contact on N-cadherin distribution. (**A**) Example of a neuron with the soma covering at least one pillar at 1 hour in culture, immunostained for N-cadherin (GC-4). Its N-cadherin crescent is oriented towards the pillar contacts. For 0.6–1 n = 112, 1.2–2 n = 115, 2.4–7 n = 67. (**B**) Example of a neuron with the soma touching the pillars 1 hour in culture. The N-cadherin crescent is in the process of being recruited towards the pillar contact.*, significantly different from random (dashed line, 50% for random positioning) using X^2^-test (p<0.05), ^#^ indicates p<0.1.For 0.6–1 n = 68, 1.2–2 n = 58, 2.4–7 n = 109. Green: CMFDA Cell tracker, Blue: Hoechst. Scale bars are 5 µm.

Recently, it has been described that in hippocampal neurons the Golgi-centrosome complex positioning is preceded by an N-cadherin crescent located at the pole of the cell from which the first neurite emerges [5]. This same sequence of events has also been observed in sensory neurons in Drosophila, where an initial N-cadherin landmark was followed by the recruitment of the centrosome [6]. We investigated whether pillar–induced polarization works via the endogenous pathway, triggering the accumulation of extracellular N-cadherin or whether an alternative route is preferred. To distinguish between these possibilities, we plated neurons onto the substrates for 1h in presence of a cell tracker (5-Chloromethylfluorescein Diacetate, CMFDA, Invitrogen). Then, we fixed the neurons and determined the position of the N-cadherin pole. In this analysis, cells positioned on the edge of a pillar bed were selected and grouped as following: (1) soma covering one or more pillars (Fig. 3A); (2) soma touching the pillars (Fig. 3B). Binning according to spacing was done in the same way as previously (dense (0.6–1 µm), intermediate (1.2–2 µm) and sparse (2.4–7 µm)). The center of the N-cadherin crescent was registered as ON when being oriented towards the pillars and OFF when away from the pillars. For a fraction of the total population (for soma covering and 0.6–1 µm: 8.9%, 1.2–2 µm: 5.7%, 2.4–7 µm: 5.6%. For soma touching and 0.6–1 µm: 6.8%, 1.2–2 µm: 7.9%, 2.4–7 µm: 9.2%) it was not possible to classify the N-cadherin crescent as ON or OFF because they were not sufficiently polarized yet, these cells were not taken into account.

From this analysis we found that the N-cadherin crescent was positioned more frequently at the side of the pillar-structured surface when the soma was covering at least one pillar (Fig. 3A, 0.6–1 µm: 65.2±4.5%, for 1.2–2 µm: 67.8±4.4%, for 2.4–7 µm: 67.2±5.7%, p<0.05 for all conditions). Under the condition that the soma only touched the pillars this trend was less evident (Fig. 3B, 0.6–1 µm: 60.3±5.9%, for 1.2–2 µm: 70.7±6.0%, for 2.4–7 µm: 58.7±4.7%. For 0.6–1 µm and 2.4–7 µm p<0.1, for 1.2–2 µm p<0.05) but still present.

### Axon Growth Preference for Neurons in Contact with Pillars

**Figure 4:**
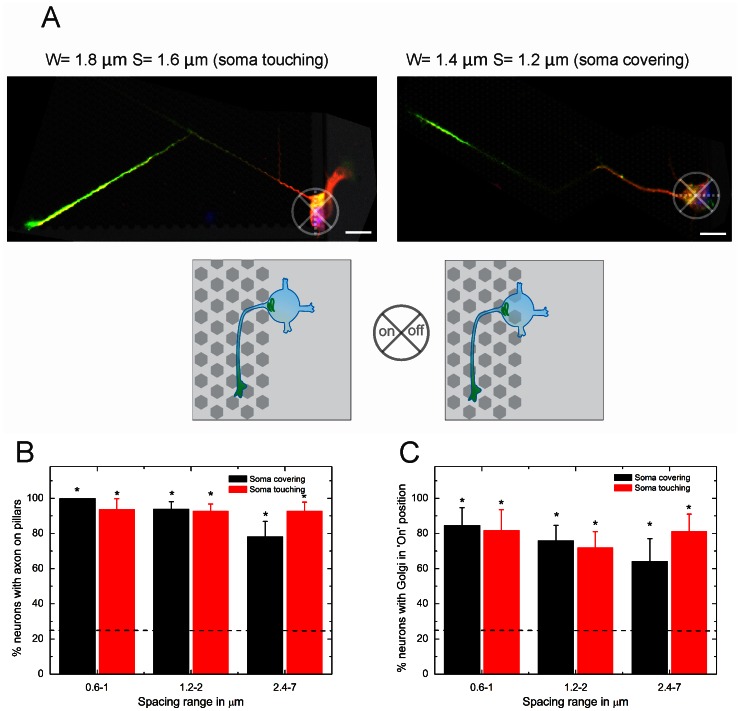
Axon position for neurons sensing topography. (**A**) Examples of axon position for neuronal soma touching the pillars (left) and covering at leat one pillar (right) (green: tau-1 axon specific staining, red: MAP-2 dendrite specific staining, blue: Hoechst). (**B**) Axon position analysis for both touching and covering conditions. (**C**) Golgi position analysis for both touching and covering conditions (dashed line, 25% for random positioning).*, significantly different from random. For ‘soma on interface’, 0.6–1 n = 16, 1.2–2 n = 32, 2.4–7 n = 23. For ‘soma touching’, 0.6–1 n = 16, 1.2–2 n = 41, 2.4–7 n = 28.

Next, we investigated whether the polarity initially induced by the topographical cues led to the axon growth preference. For this purpose, we investigated whether axonal growth was preferentially taking place on the pillar-structured surface when cells were sensing this surface (examples are shown in Fig. 4A). The results showed that a wide range of pillar spacings was able to attract the axon when the cell soma was contacting or covering the topography (Fig. 4B). For inter-pillar spacings below 2 µm, the axons were always located on the pillar bed.

We measured the position of the Golgi apparatus and quantified whether it was located at the base of the axon (Fig. 4C), as described previously [2], [7], [28]. The axis of the cell was first determined as indicated by the dotted line in Fig. 4A, after which the location of the Golgi with respect to the axon was determined. This analysis confirms that the Golgi apparatus was positioned at the base of the axon after 1 DIV.

### Effect of Pillar Dimensions on Neurite Length

**Figure 5:**
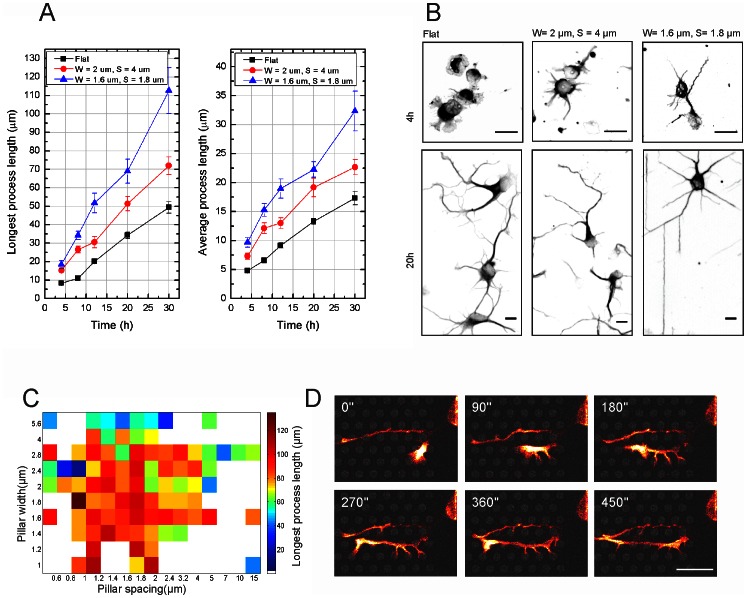
Analysis of neurite length on different pillar parameters. (**A**) Axon and average neurite length at different time points after plating. The black line shows the growth evolution on a flat substrate, red shows W = 4 µm, S = 2 µm, blue shows W = 1.8 µm, S = 1.6 µm. The process lengths for W = 1.8 µm, S = 1.6 µm, and W = 4 µm, S = 2 µm are significantly (p<0.05) higher than on flat after 4, 8, 12 and 20 h. (**B**) Examples of neuronal morphology on flat and on the previously summed pillar parameters. Scale bars are 10 µm. (**C**) Surface plot of axon length in function of width and spacing after 20 h. Maximal axon length at 20 h is achieved by pillars of W = 1–2 µm and S = 1–2 µm. (**D**) Time lapse imaging (GFP-UtrCH) of a minor neurite extending its processes. See Movie S1 for full time lapse. W = 2.8 µm, S = 1.6 µm. Scale bar is 10 µm.

To understand the relevance of quantitative variation in topographical cues (i.e. pillar diameter and spacing), neurons seeded on substrates decorated with different pillar sizes were analyzed per individual pillar bed. Neuronal architecture was identified using a neuron specific β_III_-tubulin antibody (tuj-1) and neurite lengths at different time points and on different pillar parameters were analyzed by semi-automated image analysis (Metamorph, Molecular devices, see Materials and Methods section and Fig. S1 for detailed explanation). The longest process, i.e. the axon, and the average neurite length were significantly longer compared to cells growing on a flat surface, and this already after 4h in culture (Fig. 5A, examples shown in Fig. 5B). This difference in length increased up to about two times on certain pillar dimensions after 30h in culture (axon length on W = 1.6 µm, S = 1.8 µm is 112.6±12.4 µm vs 49.3±3.2 µm on flat surface, average process length on W = 1.6 µm, S = 1.8 µm is 32.3±3.4 µm vs 17.3±1.2 µm on flat surface). Examples of neuronal morphology after 4 and 20 hours for different pillar parameters are shown in Fig. 5B. Comparing axon length for different pillar dimensions suggested that there was a range of optimal width and spacing over the whole substrate, i.e. between 1 and 2 µm for both width and spacing (Fig. 5C) that was able to generate the longest neurite lengths after 30 h in culture. Time lapse analysis of hippocampal neurons expressing a GFP-UtrCH after 1 DIV (Fig. 5D, Movie S1) showed that the actin cytoskeleton was located close to the pillars, F-actin patches were stabilized at pillar contacts.

### Effect of Pillar Dimensions on Growth Cone Area

**Figure 6:**
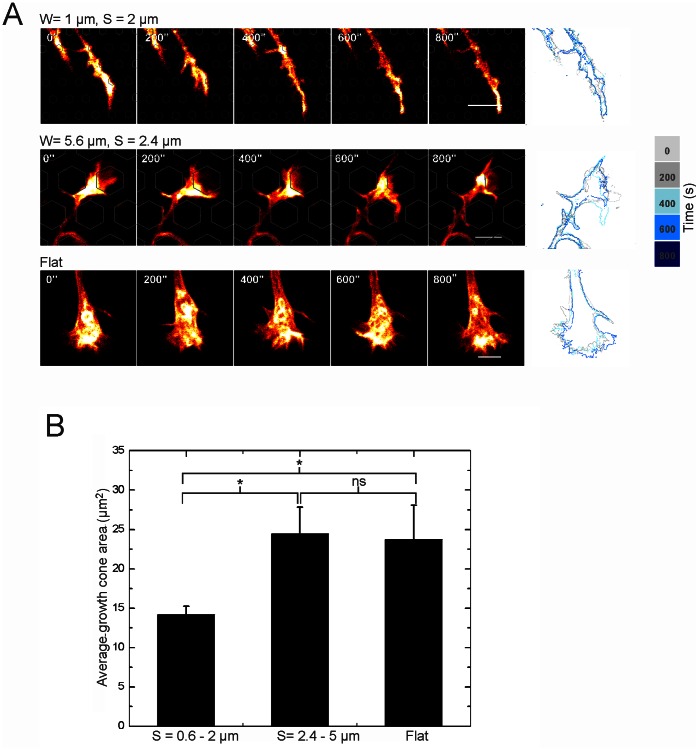
Growth cone morphology on different substrates. (**A**) Examples of time lapses of growth cones on pillar beds with different parameters. The growth cone morphology is stable over longer time (here: 800 s), confirmed by the outlined overlay images. The locations of the pillars are outlined. Scale bar is 5 µm. (**B**) Average growth cone area on different pillar spacings (Student t-test, p<0.05).

In order to gain insight into the mechanisms by which pillar contacts enhance growth, we analyzed growth cone morphology. Previous studies showed that growth cones guided by 2 µm wide adhesive tracks [31], micron sized pillars [8] and ridges [32] had a more confined morphology. Fig. 6A shows that the average growth cone size on a pillar spacing between 0.6–2 µm was significantly smaller than on a wider pillar spacing of 2.4–5 µm and on flat surfaces (Student’s t-test, p<0.05 comparing S = 0.6–2 µm vs flat and S = 0.6–2 µm vs S = 2.4–7 µm). Growth cone sizes on a spacing of 2.4–5 µm and on a flat surface were of a similar magnitude (Fig. 6B). Time lapse analysis (Fig. 6A) showed that the morphology was stable and that what we observed was not a transient state. Thus, inter-pillar spaces below 2 µm reduce the spread of the growth cone. This correlated well with the dimension range of inter-pillar spacings that lead to faster axonal growth.

### Effect of Pillar Dimensions on Tyrosine Phosphorylation Positions

Tyrosine phosphorylation is considered as one of the key steps in signal transduction and regulation of enzymatic activity and is required in a wide range of signaling pathways such as integrin signaling and focal adhesion kinase mediated actin nucleation [33]–[35]. Hence, we investigated the relationship between this biochemical modification and pillar-induced growth.

**Figure 7:**
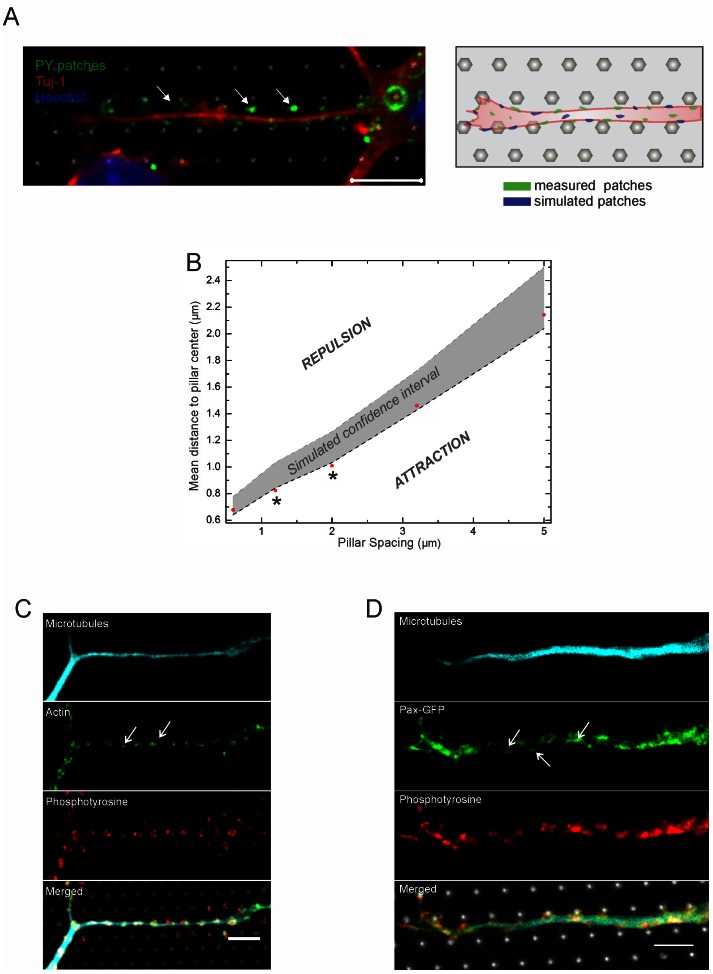
Signaling events at pillar contacts. **(**A) Example of a hippocampal neuron grown on a pillar substrate (W = 1.6 µm, S = 1.2 µm) stained for phosphotyrosine (PY, green) and tuj-1 (red). Scale bar is 5 µm. (**B**) Comparison of the mean patch distance from the pillar center (red marks) to the confidence interval for the simulated patches (gray shaded area). For a constant W = 1.6 µm, the PY patches are outside of the confidence interval for S = 1.2 µm and S = 2 µm. This is indicated by a *. See Table 1 for detailed parameter values. Individual cases are tabulated in Table S1. The y-axis values were drawn as shown in a representative example in Fig. S3B. (**C**) Co-localization of actin filaments and phosphorylated tyrosine positions. Cyan β_III_-tubulin, green actin, red phosphotyrosine. (**D**) Paxillin-GFP shows the same expression pattern than phosphotyrosine. Cyan β_III_-tubulin, green Pax-GFP, red PY. Scale bars are 5 µm.

We analyzed the co-localization of phosphotyrosine (PY) patches with pillars contact points (Fig. 7A). PY patches were attracted by contact with pillars for the spacing range between 1.2 and 2.0 µm, while this was not the case for spacings below 1.2 µm (e.g. 0.6 µm) and above 2 µm (e.g. 3.2 and 5 µm, Fig.7B).We established this result by estimating the distribution of the distances between a PY patch to the closest pillar contact (i.e. the micro-pillar underlying the neurite). This distribution was compared against the case were the PY patches were randomly distributed along the neurites’ length. Briefly, disks having the same mean area as the average PY patch were randomly placed inside the neurites projections in a number of independent Monte Carlo simulations and the nearest point-to-event-distance distribution was calculated. Detailed explanation of image processing and spatial analysis methods can be found in the Materials and Methods section and supplementary material (Fig. S2, S3). This approach was repeated for at least 11 images per pillars pacing and the confidence intervals of the distributions were computed (gray shaded area in Fig. 7B). An overview of the mean observed patch to pillar distance can be found in Table 1 (individual cases are given in Table S1). If the measured distance to the pillars center would be larger than the confidence interval of simulated distributions, this would indicate that the patches were repelled by pillar contact. On the other hand, if the measured distance to the pillar center would be smaller than the confidence interval, the PY patches would be attracted by pillar contact. The red markers in Fig. 7B indicate the measured average patch distance to pillar center. The data shows that for a pillar spacing of 0.6, 3.2 or 5 µm the mean measured distance from the PY patch to the pillar center fell within the 95% confidence interval of the simulations and hence was considered not significantly different. However, for 1.2 and 2 µm pillar spacing the patches were on average closer to the pillars than expected from the simulated cases. This indicates that particularly for the spacing range of 1–2 µm, precisely positioned growth signaling was taking place close to pillar contacts.

**Table 1:** Parameters for the construction of Fig. 7B.

Pillar Spacing (µm)	Mean distance frompatch to pillar center (µm)	Higher border simulated confidence interval (µm)	Lower border simulated confidence interval (µm)	Density of patches on neurites (%)
0.6	0.6779	0.7765	0.6402	17±6
1.2	0.8241	1.0336	0.8428	14±5
2	1.0103	1.2648	1.0334	16±6
3.2	1.461	1.7221	1.4285	18±4
5	2.1432	2.5006	2.0404	13±4

Per spacing the values for the measured mean distance of the patches to the pillar center are given, together with higher and lower border of the simulated confidence intervals. The last column shows values for the density of patches on the neurites.

F-actin showed a similar aggregation at pillar compared to PY patches, suggesting that tyrosine phosphorylation is involved in the regulation of actin remodeling (Fig. 7C). Expression of the GFP-tagged adaptor protein paxillin-GFP revealed that focal adhesion signaling and recruitment were also strongest at pillar contacts (Fig. 7D) [35]–[38]. Paxillin has recently been shown to integrate physical and chemical motility signals by determining the positions in the cell from where motile processes will form [38].

## Discussion

We report that topographic signals determine neuronal polarization from the very instant that the initial contact with the physical surface is established. First, we have shown that N-cadherin is enriched at the site of pillar contact (Fig. 3) even before the generation of the first bud (Fig. 2). From previous studies it is known that N-cadherin marks the area of initial neurite formation [5], [6]. And indeed in our experiments we also see, at a high degree of fidelity, that this initial sprout formation can be generated at the pillar contacts (Fig. 3) suggesting a conserved cellular response in reaction to external cues, both chemical and topographic. The pillar induced sprout formation behavior may well be caused by a local membrane deformation initiated by mechanical strain, which activates one or more signaling cascades [39]–[41].

Our experimental data (Fig. 2–4) showed that the N-cadherin crescent is positioned at the side of the pillar-structured surface already in round neurons. We know from previous data that N-cadherin polarizes spontaneously in neurons which are not in contact with asymmetric cues [5], and that extrinsically applied N-cadherin can position this cellular N-cadherin crescent and thus define the site from which the first neurite, i.e. the future axon, grows out [5]. However, the mechanism by which the N-cadherin crescent polarizes towards the pillars is yet unknown. We can hypothesize that either F-actin, which is concentrated at the pillar contacts (Fig. 2A, [42], [43]) or the accumulation or re-localization of focal adhesions and receptors triggered by mechanical forces [11], [21], [44]–[46] could attract N-cadherin clustering at the pillar side.

**Figure 8:**
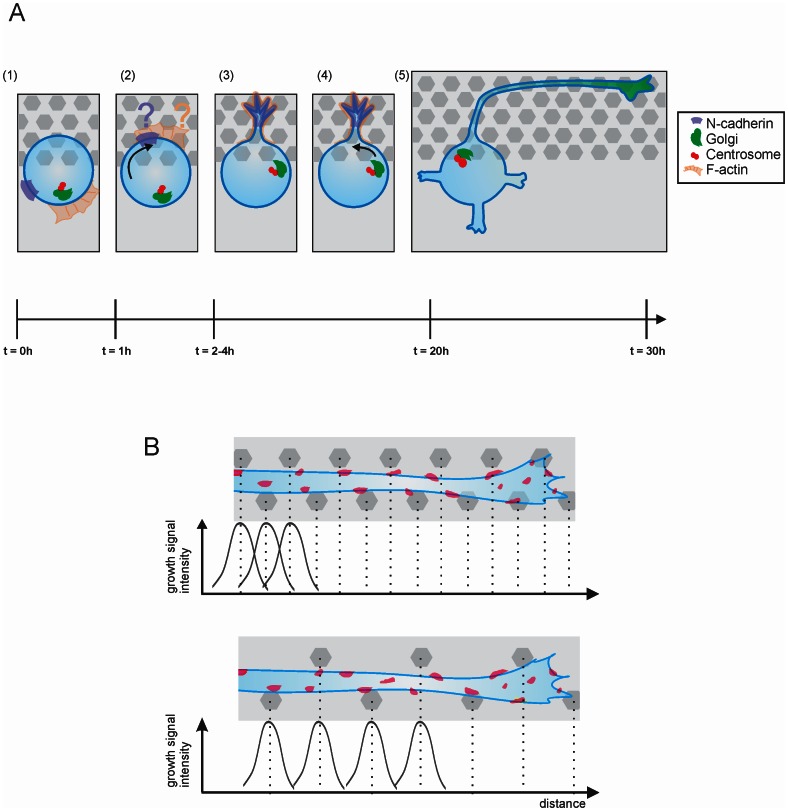
Possible model for interaction of young neurons with topography for polarization and axon formation. (**A**) When a neuron is located on the interface between a pillar array and a flat area (step 1) the N-cadherin patch is accumulated at the topographical contact, also F-actin is located at pillar contact via an unknown mechanism (step 2). After first sprout formation (step 3), the Golgi and centrosome are also recruited to the interface (step 4), from which at a later time point the axon starts to form (step 5). (**B**) Pillar contact gives rise to growth signaling, if this is repeated frequently enough during outgrowth of the neurite this leads to an overall growth encouragement. On the other hand if the pillars are spaced further apart, this neurite will not be able to exploit its full growth potential.

In any case, we can propose the following model in Fig. 8A, derived from observations of neurite formation and determination and comprising five distinct steps: when a morphologically round neuron makes contact with a pillar bed (step 1), N-cadherin (step 2) and the first bud are recruited towards the pillar contact (step 3). The Golgi apparatus and centrosome orient at the base of this neurite (step 4), and the axon arises in turn from the initial sprout (step 5). For the definition of the polarity axis and axonal identity, the pillar size and spacing dimensions are seemingly less critical, at least for the parameters used in this work. However, these parameters are more significant during axonal outgrowth. The difference in dimension-dependence of polarization and growth indicates that those events are not necessarily influenced in the same way.

During outgrowth, the pillars allow for strong and stable adhesion locations, through which neurites can exert forces, and at the same time these physical structures act as geometrical constraints providing directional guidance. The mechanical strain responsible for the initiation of the first bud enforces this sprout later on to differentiate into the axon. This occurs with a high degree of repeatability (Fig. 4).

We observed that axons grow in straight lines (Fig. 4A), similar to what has been described in the literature [41]. On our substrate straight paths are obtained by neurites growing in between pillars. The fact that some pillar dimensions (W = 1–2 µm and S = 1–2 µm) are more favorable for neurite extension (Fig. 5A–C) points towards a preferred distribution of the growth stimuli, localized at certain discrete locations. A similar dimension range has been used in other studies, not only for pillar topographies but also for grooves and ridges [8], [17], [32]. The correlation between growth cone size and outgrowth speed was described also in other neuron types [31], [32]. Growth cone filopodia sensing the environment rapidly encounter pillars that serve as anchoring points, reducing the need for a wide fan shaped growth cone. Moreover, this is likely to reduce the number of protrusion-retraction events, thus ensuring more robust neurite outgrowth compared to outgrowth on a flat substrate.

Pillar-axon contacts are enriched in highly dynamic F-actin clusters (Movie S1). In those clusters we also found an enrichment of phosphotyrosinated proteins indicating areas of high activity for growth signaling. Hence, signaling from closely spaced pillars may lead to a ‘domino’mechanism for growth stimulation, introducing boosts at pillar contacts and maintaining growth rate constant compared to the situation of more separated pillars, where signaling decay occurs (schematic model shown in Fig. 8B). Hoffman-Kim *et al.* [11] also suggested that during the process of axonal pathfinding, axons can respond to a local cue with a structural change before heading for the next permissive cue (e.g. pillar).On the other hand, as shown in Table 1, the overall density of the PY patches is not significantly different for various pillar spacing ranges, therefore the patch location seems to be of greater importance rather than the amount of patches present per neurite area. This is confirmed by Jang *et al.* (2010) in a previous study where cells were plated on ridges and were found to not contain a higher PY content than on a flat surface [32].

A major advantage of our approach of studying neuronal polarization and growth is the high reproducibility with which the substrates can be fabricated. The systematic variation of pillar parameters allows for studying changes in neuronal morphology occurring as a consequence of pillar contact. In the future topographically active surfaces can be made ‘smarter’ by introducing more electrical functionality. In addition to creating a well-organized neuronal network, this approach also allows for neuronal stimulation and electrical measurement of signals arising from single cells in a structured network with a high resolution [47], [48]. Apart from *in vitro* applications, understanding of the interface between individual neurons and topographical cues can have an impact in neuronal repair strategies [11].

To further study which molecular pathways are involved in the cytoskeletal remodeling in the response to substrate topography, the precise target proteins with tyrosine phosphorylation sites need to be identified. A myriad of tyrosine residues are phosphorylated during signaling. This is the case for e.g. paxillin [34], [49], for the actin nucleation-promoting factor N-WASP [33], and for different sites of focal adhesion kinase phosphorylated by Src [49]. These processes can also work in concert, initiated by mechanical tension that activates for instance mechanosensitive ion channels by the force neurites are exerting on the pillars while elongating [40]. Plenty of work remains to be done in order to unravel the contribution of each of these possible pathways. Since we observed that paxillin has a similar patch pattern than phosphotyrosine (Fig. 7A), we can assume that at least phosphorylation of paxillin is one of the pathways followed in signal transduction response the purely topographical input. Another unanswered question is which receptor or sensing mechanism is located upstream from the phosphorylation of tyrosine. The most likely candidate is integrin that has been shown to be mechanosensitive, a property shared with mechanosensitive ion channels and G protein-coupled receptors [11], [50]. It is interesting to note that integrin signaling is possible even without any localized ECM ligand to bind to, since we coated only with poly-L-lysine. It has been shown by Riveline et al. [51] in human fibroblasts and 3T3 cells that local application of mechanical force leads to further assembly of the existing integrin-containing molecular complex. Further, currently recognized manifestations of integrin signaling are tyrosine phosphorylation of specific proteins, increase in free intracellular calcium and cAMP level and activation of MAP and Jun kinases [51]. However, as put forward by Hoffman-Kim *et al.* [11] it is also possible that membrane distortion alone can reposition receptors and ion channels and bring them in physical proximity; consequently growth pathways could be initiated.

In conclusion, we show that topographic signaling can have a major influence on neuronal polarity establishment and maintenance. The first sprout, Golgi-centrosome position and axon specification all follow the initial N-cadherin landmark on the pillar substrate. Moreover, these events take place independently of the pillar dimension in the range we investigated. On the other hand, the quantitative outgrowth of neurites and more specifically of axons is affected by pillar dimensions. Clearly, growth cone morphology is involved in maintaining the faster outgrowth behavior, as well as precisely positioned phosphorylated tyrosine residues that are in tight contact with the pillars. All of these elements eventually lead to the pillar-mediated modeling of cytoskeletal components, such as actin and microtubules that are important in neuronal polarity.

## Materials and Methods

### Substrate Fabrication

Fabrication of the substrates was performed in a cleanroom (Class 1000) environment. To create micron scale pillar structures, first a low temperature oxide layer was deposited onto a standard 8 inch silicon wafer. After a sintering step at 455°C, standard I-line lithography was used to define areas with diameters down to 1 µm and a minimal spacing of 600 nm. A timed reactive ion etch (RIE) was performed to create pillars with a height of 3 µm and was followed by a clean with the ‘piranha’ mixture (1∶3 H_2_O_2_:H_2_SO_4_) to remove the remaining photoresist. Separate substrates were finally diced and used.

### Cell Culture, Transfection, Time Lapse Imaging

Animals were handled in accordance with international (EU Directive 86/609/EEC) and national laws governing the protection of animals used for experimental purposes, minimizing distress during procedures. The use of animals and procedures was approved by the Ethical Committee for Animal Welfare (ECD, Ethische commissie Dierenwelzijn) of KULeuven and Imec. Mouse (FVB) and rat (Wistar, Janvier) embryonic hippocampal neurons were prepared as described elsewhere [4] and plated at a density of 50 000 cells per cm^2^ on poly-L-lysine (PLL, P2636, Sigma-Aldrich, 0.5 mg/mL in borate buffer) coated substrates. In general, mouse neurons were used for all quantitative analyses, rat neurons were used for time lapse imaging with the GFP-UtrCH construct. Cells were transfected in suspension using nucleofection (Amaxa, Lonza). Neurons in the N-cadherin and first bud analysis were kept in suspensions for 2h to allow for regeneration of surface molecules which may have been destroyed during trypsin treatment used for cell dissociation. Cells used for time lapse imaging were seeded on the substrate after transfection and imaged starting from 2 h after plating until 1 DIV under a Zeiss Laser Scanning Microscope (LSM) 780 equipped with custom built live imaging set-up (Oko-lab, Italy).

### Immunocytochemistry and Morphological Analysis

Neurons were fixed in paraformaldehyde (4% PFA and 4% Sucrose in PBS) at 37°C for 10 min and permeabilized for 5 min in 0.1% Triton X-100/PBS, blocked in 2% FBS, 2% BSA, and 10% goat serum in PBS. Neurons were incubated with the primary antibody for 1 h at room temperature or at 4°C overnight. Secondary antibodies in blocking solution were added for 60 min, and were coupled to fluorophores Alexa-488, -568 and -633 (Invitrogen, Life Technologies).

The following antibodies were used: anti β_III_-Tubulin from mouse (tuj-1, Covance) or rabbit (Abcam); anti-GPP130 (Covance), and anti N-cadherin (clone GC-4, Sigma-Aldrich); anti-tau-1 (Chemicon); anti-P-Tyrosine (4G10, Upstate). Nuclei were visualized using the Hoechst compound (Hoechst 33342, Invitrogen) or propidium iodide (PI, Invitrogen). N-cadherin on the surface of neurons was detected by incubating neurons after fixation without permeabilization with an antibody recognizing a surface epitope of N-cadherin (clone GC-4, Sigma-Aldrich).

For the neurite outgrowth analysis, the semi-automated Metamorph software (Molecular Devices) was applied to analyze average and longest process length (Fig. S1). Briefly, a neurite (tuj-1) and nuclei (PI) image were loaded into the software. Based on this input the software performed a cell segmentation step (‘Segmented tuj-1 image’ and ‘Segmented PI image’). The Metamorph algorithms subsequently detected the different parameters i.e. average and longest neurite length per cell as plotted in Fig. 5A.

### Statistical Analysis

For the first sprout, Golgi and N-cadherin experiments, statistical analysis of neuron populations was performed using X^2^-test to determine a significance difference of the observed distribution compared to an expected random distribution. Growth cone areas were measured in ImageJ by default thresholding of the individual images and followed by area measurement. Groups were compared by Student’s t-tests.

### Phosphotyrosine Analysis

After the acquisition of a three channel image (substrate reflection, tuj-1 and PY) on a Zeiss LSM 780 confocal microscope, the pillar grid was isolated with a customized script in ImageJ (NIH, MA, USA) and the tuj-1 and PY images were thresholded (cfr. Methods S1 and Fig. S2, B: ‘Grid image’, C: ‘Tuj-1 image’, E: ‘Segmented PY patches’). Cell bodies were excluded from this analysis to avoid bias. Randomly positioned blobs having the same average diameter as determined from the average of a statistical sample of measured PY patches were randomly placed on the thresholded tuj-1 image (Fig. S2D, ‘Simulated PY patches’).

The spatial distribution of PY patches was revealed by calculation of the ‘nearest point to event distance’ followed by comparison to a reference random distribution comprising the null hypothesis H_0_. The method is described in greater detail elsewhere [52]. Briefly, the mapped distribution of events (the segmented PY image) can be recast into the conceptual framework of spatial point processes in the plane. In the framework of hypothesis testing, observed patterns of patches can be compared against realizations of various types of stochastic spatial point processes (Fig. S3A–C). A benchmark for such comparisons is the homogeneous Poisson process in the plane because it is the simplest and the best-studied spatial process. This process is characterized with *complete spatial randomness* having the properties of (i) homogeneity–the intensity of the process is constant over the region of study, and (ii) spatial independence in the occurrence of the events–numbers of the observed points in neighboring regions do not correlate with each other.

In our approach, the locations of the patches (i.e. ‘events’) are referenced to the immutable hexagonal grid of pillars on the substrate (i.e. ‘observation points’, see Fig. S3A). The distribution of nearest point to event distances is denoted conventionally by 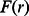. Under CSR in the undoubted plane.
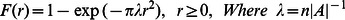
(1)


Here is the number of events in 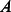, the region of interest, in this case the tuj-1 thresholded image. However, in our measurements we were confronted with a multitude of disjoint regions representing the neurites’ projections (Fig. S2). In such circumstances, the distribution of is intractable, therefore for making statistical inferences one needs to resort to Monte Carlo simulations. We addressed this challenge by realizing instances of homogeneous Poisson process bound to the regions of neurites (patched CSR, pCSR) as described in [53]. The simulations were performed as following: disks having the same average area as the observed patches were randomly placed on the segmented neurite mask (i.e. projections, Fig. S2C), the distance to the nearest point on grid was identified and an empirical distribution was computed for every analyzed image.

Fig. S3B shows an example for S = 2 µm of the observed data 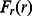 that was compared to the distribution 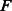 under pCSR. Following Diggle (1983) [52], a significance test was introduced as described in Prodanov (2007) [53]. More detailed explanation and equations can be found in Methods S1. Lower and upper confidence interval boundaries were estimated from 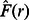 for every image (An example is shown in Fig. S3D). If a confidence interval boundary is considered as a random variable then following the Central Limit Theorem the sample average of such variable will reflect its population value.

Finally the mean of the measured, segmented images was compared to the 95% confidence interval boundaries that were taken as significance levels. To obtain a statistical inference per condition all such intervals were aggregated and averaged. The final result for all investigated spacings is shown in Fig. 7B (lower region in the plot is ‘attraction’ (i.e. the patches are closer to the pillar than under CSR) while the upper region is ‘repulsion’ (i.e. the patches are further from the pillar than under CSR).

## Supporting Information

Figure S1 **Workflow of morphometric analysis by Metamorph analysis and segmentation.** An example of tuj-1 and PI image of neurons at 20 h in culture on pillars of W = 1 µm, S = 1 µm used for Metamorph neurite outgrowth analysis. In the segmentation image each cell with its neurites is labeled by a different color.
(TIF)

Figure S2 **Workflow of image processing and simulation steps.** (**A**) Example of a composite confocal image with microtubules stained in red, PY patches in green and nuclei in blue, gray in reflectance. (**B**) shows the thresholded gray channel grid, indicating the individual pillar centers. (**C**) The tuj-1 thresholded image where the cell bodies are cut out. (**D**) The simulated PY patches shown in cyan on the tuj-1 image and (**E**) the segmented PY patches. (**F**) The histogram of patch areas for this particular example, the mean patch size is 0.331±0.002 µm^2^ (mean ± sem).
(TIF)

Figure S3
**Workflow of the Monte Carlo statistical analysis.** (**A**) On the schematic the outline of the neurites is shown in black, the pillar centers are shown in gray, simulated patches in cyan and observed PY patches in dark blue. (**B**) Detail of schematic shown in (A). With a nearest neighbor algorithm the closest patch-to-pillar distances were computed for both the simulated and observed patches (r_1s_ vs r_2s_ for the simulated and r_1r_ vs r_2r_ for the observed patches). (**C**) The pillar centers are ‘points’ and the patches (observed or simulated, indicated by a star) are ‘events’. The nearest point to event was determined as 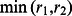 for both simulated and measured patches. (**D**) An example for W = 1.6 µm and S = 2 µm of the cumulative distribution functions (cdf) used for constructing Fig. 7B. At the 50% level of the cdf, the corresponding distances for the observed data, lower and upper confidence interval were eventually plotted on the y-axis of Fig. 7B vs the spacing for which the analysis was performed.(TIF)

Table S1 **Detailed data of the individual analyzed images.** Per spacing at least 11 images were analyzed, the real and simulated mean distances to the pillar center are given per image as well as the calculated p-values.
(DOCX)

Methods S1 **Supplementary Methods.**
(DOCX)

Movie S1 **Behavior of neurites on a pillar bed.** Hippocampal neuron (GFP-UtrCH) at 1 DIV growing on pillar substrate. Full time lapse of fragment shown in Fig. 5D. White arrows indicate actin patches enriched at pillar contacts. Green arrow position shows the minor neurite extending filopodia to the pillars when extending. 90 s per frame. W = 2.8 µm, S = 1.6 µm at the left side of the ridge, W = 2.8 µm, S = 1.8 µm at the right side. Scale bar is 10 µm.
(AVI)
