# Supplementary figures and images for: Substrate Topography Determines Neuronal Polarization and Growth In Vitro
Source: PLoS One. 2013 Jun 13;8(6):e66170. doi: 10.1371/journal.pone.0066170 (PMC3681759; doi:10.1371/journal.pone.0066170)

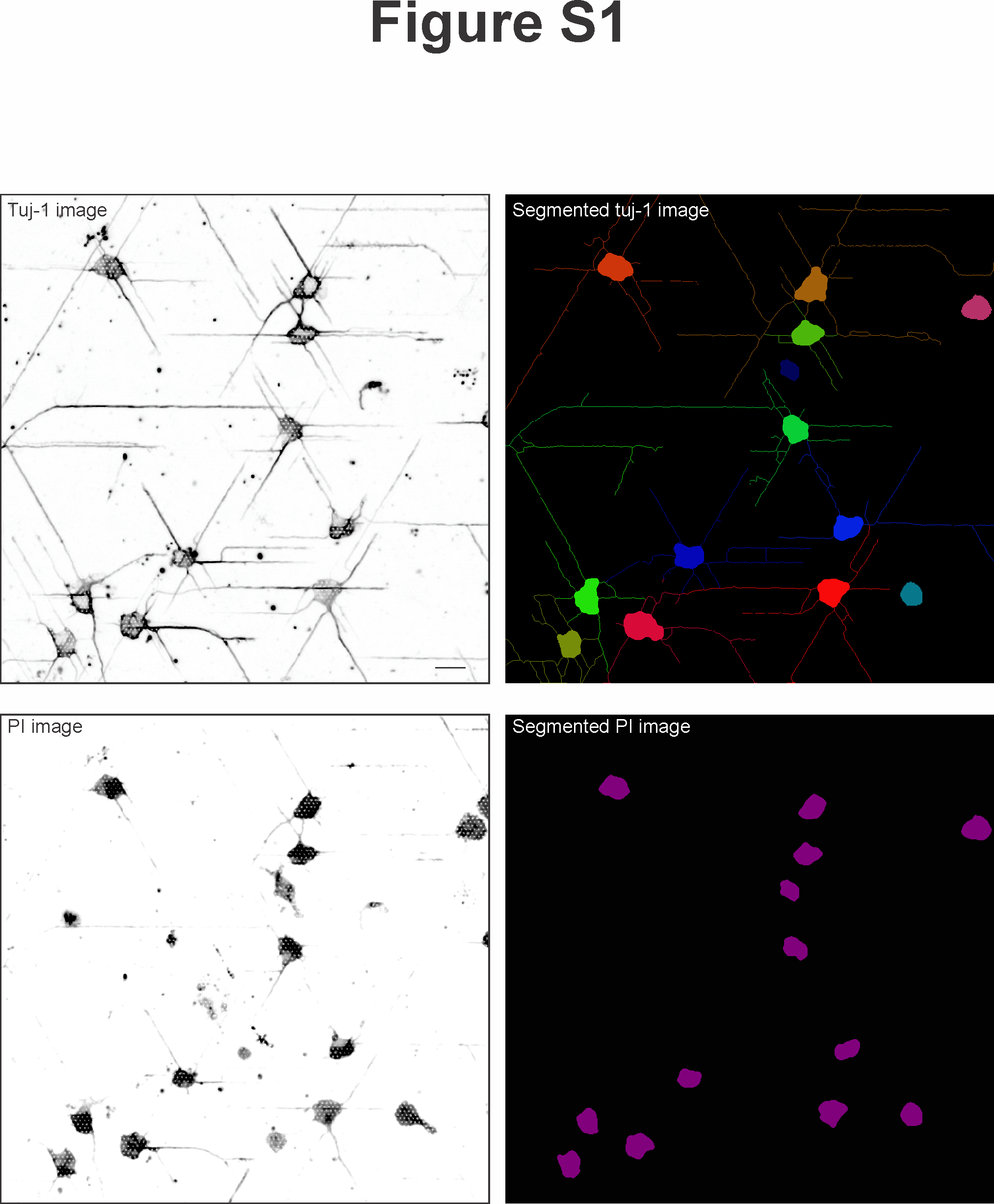

Supplement: Figure S1 — Workflow of morphometric analysis by Metamorph analysis and segmentation. An example of tuj-1 and PI image of neurons at 20 h in culture on pillars of W = 1 µm, S = 1 µm used for Metamorph neurite outgrowth analysis. In the segmentation image each cell with its neurites is labeled by a different color. (TIF) [file pone.0066170.s001.tif]

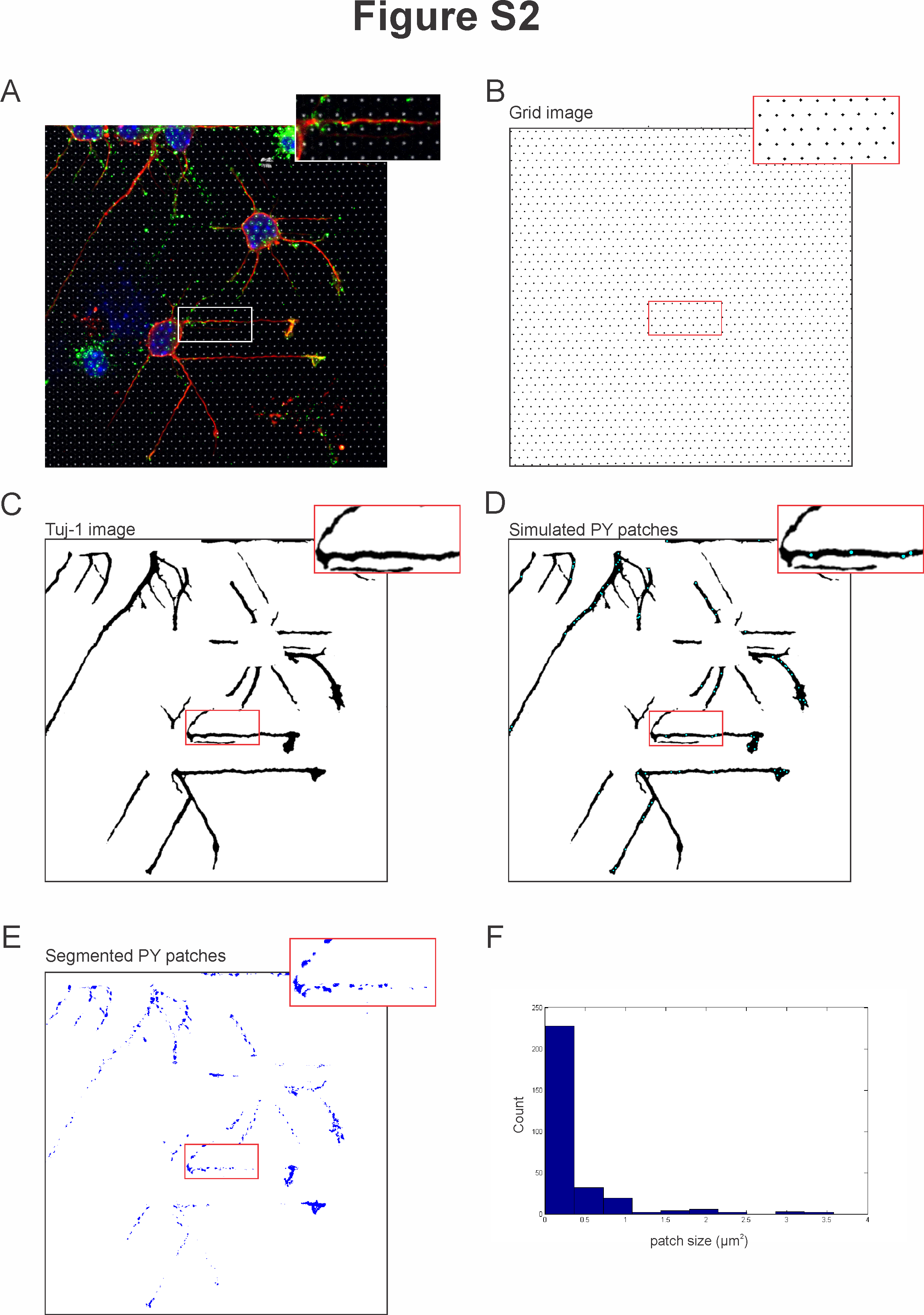

Supplement: Figure S2 — Workflow of image processing and simulation steps. (A) Example of a composite confocal image with microtubules stained in red, PY patches in green and nuclei in blue, gray in reflectance. (B) shows the thresholded gray channel grid, indicating the individual pillar centers. (C) The tuj-1 thresholded image where the cell bodies are cut out. (D) The simulated PY patches shown in cyan on the tuj-1 image and (E) the segmented PY patches. (F) The histogram of patch areas for this particular example, the mean patch size is 0.331±0.002 µm2 (mean ± sem). (TIF) [file pone.0066170.s002.tif]

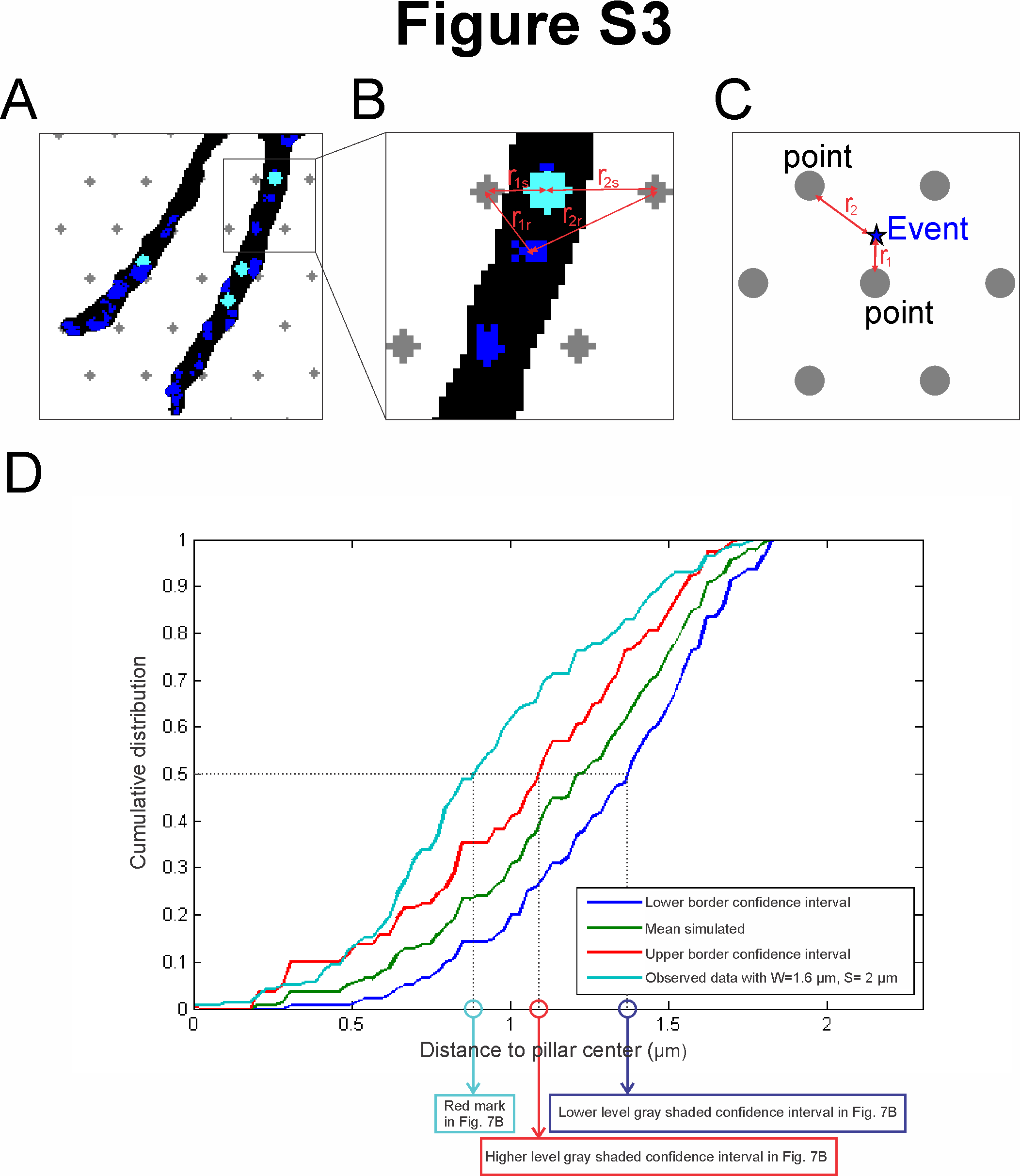

Supplement: Figure S3 — Workflow of the Monte Carlo statistical analysis. (A) On the schematic the outline of the neurites is shown in black, the pillar centers are shown in gray, simulated patches in cyan and observed PY patches in dark blue. (B) Detail of schematic shown in (A). With a nearest neighbor algorithm the closest patch-to-pillar distances were computed for both the simulated and observed patches (r1s vs r2s for the simulated and r1r vs r2r for the observed patches). (C) The pillar centers are ‘points’ and the patches (observed or simulated, indicated by a star) are ‘events’. The nearest point to event was determined as for both simulated and measured patches. (D) An example for W = 1.6 µm and S = 2 µm of the cumulative distribution functions (cdf) used for constructing Fig. 7B. At the 50% level of the cdf, the corresponding distances for the observed data, lower and upper confidence interval were eventually plotted on the y-axis of Fig. 7B vs the spacing for which the analysis was performed. (TIF) [file pone.0066170.s003.tif]
