# Supplementary material for: Substrate Topography Determines Neuronal Polarization and Growth In Vitro
Source: PLoS One. 2013 Jun 13;8(6):e66170. doi: 10.1371/journal.pone.0066170 (PMC3681759; doi:10.1371/journal.pone.0066170)
Supplement: Table S1 — Detailed data of the individual analyzed images. Per spacing at least 11 images were analyzed, the real and simulated mean distances to the pillar center are given per image as well as the calculated p-values. (DOCX) [file pone.0066170.s004.docx]

**Supplementary Table**

**Table S1: Detailed data of the individual analyzed images.**

| Image number | | Spacing (um) | | Real mean distance to pillar center | | Simulated mean distance to pillar center | p-value | |
| --- | --- | --- | --- | --- | --- | --- | --- | --- |
| 1 | | 0.6 | | 0.723 | | 0.786 | 0.0356 | |
| 2 | | 0.6 | | 0.581 | | 0.707 | 0.0598 | |
| 3 | | 0.6 | | 0.723 | | 0.786 | 0.0233 | |
| 4 | | 0.6 | | 0.707 | | 0.754 | 0.0033 | |
| 5 | | 0.6 | | 0.790 | | 0.817 | 0.0133 | |
| 6 | | 0.6 | | 0.801 | | 0.786 | 0.1296 | |
| 7 | | 0.6 | | 0.723 | | 0.723 | 0.5615 | |
| 8 | | 0.6 | | 0.707 | | 0.707 | 0.0532 | |
| 9 | | 0.6 | | 0.754 | | 0.707 | 0.0897 | |
| 10 | | 0.6 | | 0.644 | | 0.754 | 0.0066 | |
| 11 | | 0.6 | | 0.644 | | 0.707 | 0.0963 | |
| 12 | | 0.6 | | 0.723 | | 0.723 | 0.3289 | |
| 1 | | 1.2 | | 0.860 | | 1.080 | 0.0033 | |
| 2 | | 1.2 | | 0.900 | | 1.020 | 0.0033 | |
| 3 | | 1.2 | | 0.860 | | 1.020 | 0.0033 | |
| 4 | | 1.2 | | 0.900 | | 0.940 | 0.0033 | |
| 5 | | 1.2 | | 0.800 | | 1.000 | 0.0033 | |
| 6 | | 1.2 | | 0.920 | | 0.940 | 0.01 | |
| 7 | | 1.2 | | 0.860 | | 1.000 | 0.0033 | |
| 8 | | 1.2 | | 0.860 | | 1.060 | 0.0033 | |
| 9 | | 1.2 | | 0.900 | | 0.980 | 0.0066 | |
| 10 | | 1.2 | | 0.860 | | 1.020 | 0.0166 | |
| 11 | | 1.2 | | 0.820 | | 0.980 | 0.0033 | |
| 12 | | 1.2 | | 0.700 | | 0.900 | 0.0033 | |
| 13 | | 1.2 | | 0.780 | | 0.900 | 0.0033 | |
| 1 | | 2 | | 1.003 | | 1.389 | 0.01 | |
| 2 | | 2 | | 0.951 | | 1.286 | 0.0033 | |
| 3 | | 2 | | 1.003 | | 1.311 | 0.0033 | |
| 4 | | 2 | | 0.900 | | 1.234 | 0.0033 | |
| 5 | | 2 | | 1.260 | | 1.311 | 0.2425 | |
| 6 | | 2 | | 1.054 | | 1.209 | 0.01 | |
| 7 | | 2 | | 1.054 | | 1.286 | 0.0033 | |
| 8 | | 2 | | 1.209 | | 1.311 | 0.0133 | |
| 9 | | 2 | | 1.054 | | 1.311 | 0.0033 | |
| 10 | | 2 | | 0.771 | | 0.926 | 0.0033 | |
| 11 | | 2 | | 0.823 | | 0.823 | 0.0033 | |
| 1 | 3.2 | | 1.646 | | 1.611 | | | 0.1063 |
| 2 | 3.2 | | 1.509 | | 1.646 | | | 0.0033 |
| 3 | 3.2 | | 1.509 | | 1.611 | | | 0.2259 |
| 4 | 3.2 | | 1.406 | | 1.611 | | | 0.01 |
| 5 | 3.2 | | 1.783 | | 1.714 | | | 0.1429 |
| 6 | 3.2 | | 1.303 | | 1.611 | | | 0.0033 |
| 7 | 3.2 | | 1.131 | | 1.406 | | | 0.0498 |
| 8 | 3.2 | | 1.166 | | 1.371 | | | 0.0033 |
| 9 | 3.2 | | 1.269 | | 1.406 | | | 0.3787 |
| 10 | 3.2 | | 0.994 | | 1.509 | | | 0.0033 |
| 11 | 3.2 | | 1.406 | | 1.646 | | | 0.0033 |
| 12 | 3.2 | | 1.474 | | 1.714 | | | 0.0033 |
| 13 | 3.2 | | 1.646 | | 1.851 | | | 0.0033 |
| 14 | 3.2 | | 1.817 | | 1.714 | | | 0.0465 |
| 15 | 3.2 | | 1.611 | | 1.749 | | | 0.0033 |
| 16 | 3.2 | | 1.611 | | 1.714 | | | 0.0033 |
| 1 | 5 | | 2.074 | | 2.498 | | | 0.0033 |
| 2 | 5 | | 2.498 | | 2.640 | | | 0.0631 |
| 3 | 5 | | 2.216 | | 2.263 | | | 0.3555 |
| 4 | 5 | | 2.121 | | 2.121 | | | 0.5415 |
| 5 | 5 | | 2.404 | | 1.404 | | | 0.4452 |
| 6 | 5 | | 1.980 | | 2.404 | | | 0.0033 |
| 7 | 5 | | 2.169 | | 2.074 | | | 0.2691 |
| 8 | 5 | | 2.263 | | 2.357 | | | 0.0698 |
| 9 | 5 | | 2.310 | | 2.121 | | | 0.0332 |
| 10 | 5 | | 2.310 | | 2.357 | | | 0.3688 |
| 11 | 5 | | 2.498 | | 2.498 | | | 0.0033 |
| 12 | 5 | | 2.074 | | 2.404 | | | 0.0133 |
|  |  | |  | |  | | |  |
