# Supplementary material for: Substrate Topography Determines Neuronal Polarization and Growth In Vitro
Source: PLoS One. 2013 Jun 13;8(6):e66170. doi: 10.1371/journal.pone.0066170 (PMC3681759; doi:10.1371/journal.pone.0066170)
Supplement: Methods S1 — Supplementary Methods. (DOCX) [file pone.0066170.s005.docx]

**Substrate Topography Determines Neuronal Polarization and Growth *in vitro***

: **Supporting information**

**Supplementary Methods**

Phosphotyrosine analysis

The following algorithm was used for the analysis of the PY patches (ImageJ/Matlab). This is applied to the specific example shown in Fig. S2. The image as acquired from the microscope is shown in Fig. S2A. After separation of the channels there are five individual images: (i) background (grid) image showing the pillars, (ii) Hoechst image, (iii) tuj-1 image, (iv) PY image. The following steps are performed in ImageJ with custom written plug-ins:

- Segmentation of the pillar image to obtain Fig. S2B
  1. Perform morphological opening with a circle radius of 2 pixels
  2. Perform morphological closing with a circle radius of 2 pixels
  3. Auto threshold step
  4. Perform ‘maximum entropy’ threshold step
  5. Convert to mask
  6. Make binary
  7. Generate the ultimate eroded points
  8. Perform morphological dilation with a circle radius of 2 pixels
  9. Analyze particles with a size of 0-infinity and circularity of 0-1
- Simulating patches in the tuj-1 image to obtain Fig. S2C and D
  1. Perform ‘default dark’ threshold step
  2. Manually cut out cell bodies and possible debris (final result Fig. S2C)
  3. Simulate patches (Example shown in Fig. S2D)
     1. Radius of the seed (simulated patch) = 5 pixels,
     2. The total area of patches per simulation cannot exceed 5% of the total neurite area
     3. Repeat simulation 100 times
- Distribution of PY patches to obtain Fig. S2E
  1. Threshold PY image (70,255)
  2. Select all patches located inside of the neurites for analysis (‘AND’ command in Image Calculator)
  3. Perform morphological closing with a circle radius of 2 pixels
  4. Analyze particles with a size of 0.01-4 µm^2^ and circularity of 0-1
     1. The area of each PY patch is measured and analyzed. For this example the histograms of analyzed patches (patch area vs count) is shown in Fig. S2F. In this case the mean PY patch area is 0.331 ± 0.002 µm² (mean ± sem).

An example is shown in Fig. S3A-C; in the neurite dark blue areas are the observed patches, the cyan areas are the simulated. All the results tables were then loaded into Matlab with the Matlab-ImageJ interface module MIJ []. We computed the distance of a patch to the nearest pillar (Fig. S3A; comparing r_1_ and r_2_ for both observed and simulated data points. Objects were represented by their top left starting point.) This process was performed for every point, after which we assembled a distance histogram (Fig. S2F), an empirical estimator of the cumulative distribution function.

The cumulative distribution functions (i.e. F-functions) of (i) the simulated and (ii) measured patch distributions vs the grid were computed. An example is given in Fig. S3D, where the median is outlined and the 95% confidence intervals (upper in red, lower in blue) of the simulated patch distribution are displayed.

Following Diggle (1983) [], a significance test was introduced as described in Prodanov (2007) [], the integrals

$u_{i}=\int_{0}^{R_{\max}} \left[H_{i}(r)-\frac{1}{N_{s}-1}\sum_{j=1}^{N_{s}-1} H_{j}j(r)\right]^{2}dr$ Eq. (2)

are calculated and ranked, the p-value is

$p=2\left[1-\frac{rank(u_{observed})}{N_{s}}\right]$ Eq. (3)

Lower and upper confidence interval boundaries were estimated from $\hat{F}(r)$for every image. Finally, to obtain a statistical inference per condition all such intervals were aggregated and averaged. These final data points are plotted for each investigated spacing in Fig. 7B.

**Supplementary references**

[1] Sage D, Prodanov D, Tinevez JY, Schindelin J (2012) Daniel sage: MIJ: Making interoperability between imagej and matlab possible. In: ImageJ User & Developer Conference (IUDC’12). Mondorf-les-Bains, Grand Duchy of Luxembourg. http://­bigwww.epfl.ch/­publications/­sage1205.html.

[2] Diggle P (1983) Statistical analysis of spatial point patterns. London: Academic press. : 148 p.

[3] Prodanov D, Nagelkerke N, Marani E (2007) Spatial clustering analysis in neuroanatomy: applications of different approaches to motor nerve fiber distribution. J Neurosci Methods 160: 93–108.
